# Establishment of a novel nomogram for the clinically diagnostic prediction of minimal change disease, −a common cause of nephrotic syndrome

**DOI:** 10.1186/s12882-020-02058-3

**Published:** 2020-09-14

**Authors:** Gaofei Yan, Guanzhi Liu, Xuefei Tian, Lifang Tian, Hao Wang, Peiyao Ren, Xiaotao Ma, Rongguo Fu, Zhao Chen

**Affiliations:** 1 Department of Nephrology, The Second Affiliated Hospital of Xi’an Jiaotong University, Xi’an, 710005 China; 2 Bone and Joint Surgery Center, The Second Affiliated Hospital of Xi’an Jiaotong University, Xi’an, 710005 China; 3 Department of Internal Medicine, Yale University school of Medicine, New Haven, CT 06520 USA

**Keywords:** Minimal change disease, Noninvasive, Predict, Multiparameter analysis

## Abstract

**Background:**

Minimal change disease (MCD) is one of the major causes of nephrotic syndrome (NS). A confirmed MCD diagnosis mainly depends on renal biopsy at present, which is an invasive procedure with many potential risks. The overall incidence of complications caused by renal biopsy procedures has been reported as approximately 11 and 6.6% outside and within China, respectively. Unfortunately, there is currently no noninvasive procedure or practical classification method for distinguishing MCD from other primary glomerular diseases available.

**Method:**

A total of 1009 adult patients who underwent renal biopsy between January 2017 and November 2019 were enrolled in this study. Twenty-five parameters extracted from patient demographics, clinical manifestations, and laboratory test results were statistically analysed. LASSO regression analysis was further performed on these parameters. The parameters with the highest area under the curve (AUC) were selected and used to establish a logistic diagnostic prediction model.

**Results:**

Of the 25 parameters, 14 parameters were significantly different (*P* < 0.05). MCD patients were mostly younger (36 (22, 55) vs. 41 (28.75, 53)) and male (59% vs. 52%) and had lower levels of diastolic blood pressure (DBP) (79 (71, 85.5) vs. 80 (74, 89)) and IgG (5.42 (3.17, 6.36) vs. 9.38 (6.79, 12.02)) and higher levels of IgM (1.44 (0.96, 1.88) vs. 1.03 (0.71, 1.45)) and IgE (160 (46.7, 982) vs. 47.3 (19, 126)) than those in the non-MCD group. Using the LASSO model, we established a classifier for adults based on four parameters: DBP and the serum levels of IgG, IgM, IgE. We were able to clinically classify adult patients with NS into MCD and non-MCD using this model. The validation accuracy of the logistic regression model was 0.88. A nomogram based on these four classifiers was developed for clinical use that could predict the probability of MCD in adult patients with NS.

**Conclusions:**

A LASSO model can be used to distinguish MCD from other primary glomerular diseases in adult patients with NS. Combining the model and the nomogram potentially provides a novel and valuable approach for nephrologists to diagnose MCD, avoiding the complications caused by renal biopsy.

## Background

Minimal change disease (MCD) is one of the major causes of nephrotic syndrome (NS), which commonly occurs in senior people and children. The pathogenesis of MCD is not quite clear, but it is likely that its mechanisms are not the same for patients in different age subgroups. The role of podocytes in proteinuria development in MCD has received increasing attention in the last decade [1, 2]. MCD is the most common cause of NS in children greater than 1 year of age, accounting for 70–90% of patients. In NS patients at puberty, this proportion of MCD significantly decreases as other glomerular diseases, such as membranous nephropathy, become more common causes [3, 4]. MCD can be found in approximately 10–15% of adult patients with NS [5, 6], and is the cause of idiopathic NS in 90% of children. As such, for children with idiopathic NS, treatment is usually initiated without the need for renal biopsy unless clinical and laboratory evidence suggests other glomerular diseases. The causes of NS in adults are more varied and renal biopsy is usually required for diagnosis [7]. The diagnosis of MCD in adults currently mainly relies on renal biopsy.

The advantages of renal biopsy are its relatively safety, simplicity, and ease with which it is performed; however, the invasive procedure is not risk-free [8]. The most frequent clinically significant complications following percutaneous renal biopsy include haemorrhage, arteriovenous shunting, infection, nephrectomy and even death in some rare conditions. Additionally, renal biopsies cannot be applied to patients with NS for which it is contraindicated [9, 10] or those who refuse the procedure, while certain hospitals lack nephrologists with sufficient operative skills to perform the biopsy. Therefore, it is necessary and important to explore a non-invasive and practical classification model for distinguishing MCD from non-MCD for adult patients with NS.

It is often quite difficult to select appropriate predictors for such a prognostic model and estimate the regression coefficients for the selected predictors correctly. The traditional method for variable choice is stepwise regression, but the resulting model often has high variance and poor flexibility. In recent years, the introduction of the concept of regularized regression has been a critical breakthrough in the regression analysis field, in which the most well-recognized and widely used method is LASSO regression, proposed in 1996.

LASSO regression sets relatively unimportant independent variable coefficients to zero, which are then excluded from modelling by constructing a penalizing function for all variable coefficients [11, 12]. Its clinical applications are broadly used for supervised learning, which starts with a goal for predicting a known output or target. Supervised learning focuses on classification, which involves choosing one among several subgroups to best describe a new data instance, and prediction which involves estimating an unknown parameter. Supervised learning is also often used to estimate risk. After statisticians’ unremitted efforts for more than 20 years, initially starting from the LASSO concept proposed by R. Tibshirani in 1996 [13] to now, a relatively complete set of theories has been established. All of these factors have made statistical inferences based on punishment estimates and the establishment of our diagnostic prediction model for MCD a reality.

To the best of our knowledge, we are the first to have developed a non-invasive diagnostic model using LASSO logistic regression and determined markers for evaluating disease severity by analysing serological parameters and clinical signs in the present study. Moreover, we assessed the predictive accuracy of this model in internal patient test groups. The establishment of the model could provide physicians with an additional tool for clinical diagnostic evaluation of adult patients with NS, especially when renal biopsy is impractical for various reasons.

## Method

### Patients and clinical information

A total of 1009 inpatients with NS from January 12,017 to November 52,019 in the Department of Nephrology, the Second Affiliated Hospital of Xi’an Jiaotong University were enrolled in this study. Among them, there were 71 patients with MCD and 938 patients with non-MCD confirmed by renal biopsy.

The inclusion criteria were as follows: a) renal biopsy performed during hospitalization and the lack of a prior renal pathologic diagnosis from other hospital, b) lack of any immunosuppressive treatment or renal replacement therapy before admission, and c) age between 14 and 75 years. Patients meeting any of the following criteria were excluded: a) no renal biopsy reports in our hospital, b) reports of any immunesuppression treatment or renal replacement therapy before hospitalization, c) ages younger than 14 years, and d) a majority of clinical data missing.

### Definition

The kidney specimen for renal pathological diagnosis was taken by ultrasound-guided percutaneous renal biopsy, which was performed before administration of immunosuppressive therapy. The specimens were examined under light microscopy and transmission electron microscopy in accordance with standard procedures and were reviewed by more than two experienced renal pathologists. The renal pathological features of MCD are as follows: no glomerular lesions or only minimal mesangial prominence examined by light microscopy; negative staining or low-intensity staining for C3 and IgM examined by immunofluorescence microscopy; and diffuse foot process effacement without electron-dense deposits determined by transmission electron microscopy [5]. The non-MCD renal pathological diagnoses for patients with NS included mesangial proliferative glomerulonephritis (MsPGN), focal segmental glomerulosclerosis (FSGS), membranous nephropathy (MN), and membranoproliferative glomerulonephritis (MPGN).

### Statistical analysis

Statistical differences between 2 groups were evaluated by t-tests, Mann-Whitney U tests, chi-square tests, logistic LASSO regression analysis and receiver operating characteristic (ROC) analysis as appropriately described below. Statistical analysis was performed by IBM SPSS Statistics 20 software. Normally distributed data are expressed as the mean ± standard deviation (SD) and were evaluated using unpaired Student’s t tests. Non-normally distributed data are expressed as medians with corresponding 25th and 75th percentiles (interquartile range) and were compared using Mann-Whitney U tests. Categorical variables were analysed using chi-square tests. Logistic LASSO regression analysis was performed to establish the diagnostic model. ROC analysis was performed to measure the diagnostic value of clinical factors and an area under the ROC curve (AUC) greater than 0.70 was considered to indicate good specificity [14]. A *P* value less than 0.05 was considered statistically significant.

### Model selection and validation

Due to the limited size of the data, we choose cross-validation for model selection. Cross-validation divides the obtained data multiple times into different training sets and validation sets. If the prediction model established with the training set shows the corresponding characteristics (discrimination and consistency) on the validation set, it means that the established prediction model works well.

First, we randomly divided the sample data into two parts (75% training set, 25% validation set), then used the training set to train the model and verified the model and parameters with the validation set. Of course, the validation set is only a quarter of the original data. This validation set is considered internal verification. Then, we resampled the data to generate additional training sets and validation sets and repeated the training and validation steps. Finally, we selected the optimal model and parameters.

### Procedures

LASSO regression analysis of the data was performed with the “glmnet” package in R software. The variables that achieved the highest AUC in the LASSO regression model were selected and used to construct the logistic diagnostic model, followed by establishment of the nomogram with the “RMS” package. Seventy-five percent of the data were selected randomly as the training set, while the remaining 25% of the data were assigned as the validation set. Then, we assessed how close the actual outcomes were able to be accurately predicted by every nomogram. ROC analysis was carried out by the R package “pROC”.

## Results

### Patient characteristics

Table 1 shows detailed clinicopathological characteristics and differences between the MCD patients and non-MCD patients. Data are displayed as the median (interquartile range) except sex. There were 14 clinical indicators with significant differences between the two groups, including systolic blood pressure (SBP), diastolic blood pressure (DBP), haemoglobin (HB), platelets (PLT), red blood cell (RBC) count, immunoglobulin G (IgG), immunoglobulin A (IgA), immunoglobulin M (IgM), complement 3 (C3), complement 4 (C4), immunoglobulin E (IgE), complement 1q (C1q), urine protein and eGFR (*P* < 0.05). The median values of blood pressure in the non-MCD group, including SBP and DBP, were higher than those in the MCD group. Interestingly, although the difference in blood pressure between the MCD group and the non-MCD group was statistically significant, only slight differences in the medians between these two groups were observed (121/79 mmHg vs. 125/80 mmHg), and the blood pressure levels of most patients were within the normal range.

**Table 1:** Baseline patient characteristics and differences between the MCD group and non-MCD group

Characteristics [median (Q1, Q3)]	TOTAL (*n* = 1009)	MCD (*n* = 71)	Non-MCD (*n* = 938)	Z	*P* value
Age (years)	41 (28,53)	36 (22,55)	41 (28.75,53)	− 1.193	0.2
Sex (male/female), n	532/477	42/29	490/448	**/**	**/**
SBP (mmHg)	124 (114,137)	121 (109,132)	125 (114,138)	−2.097	0.04 *
DBP (mmHg)	80 (73,89)	79 (71,85.5)	80 (74,89)	−2.223	0.03 *
Height (cm)	165 (160,172)	166 (163,172)	165 (160,172)	−0.891	0.4
Weight (kg)	65 (56,75)	63.25 (55.25,75.5)	65 (56,75)	−0.604	0.5
BMI (kg/m^2^)	23.705 (20.960,26.515)	23.63 (20.52,27.05)	23.73 (21,26.4)	−0.263	0.8
Glu (mmol/L)	4.81 (4.39,5.39)	4.625 (4.19,5.075)	4.83 (4.41,5.4)	−1.898	0.06
WBC (*10^9^ /L)	6.37 (5.18,7.89)	6.52 (4.84,7.98)	6.36 (5.19,7.89)	−0.227	0.8
HB (g/L)	133 (116,147)	142 (133,155)	132 (115,146)	−4.578	<0.001 *
PLT (*10^9^ /L)	224 (179,269)	253.5 (212.5293.25)	221 (177.5266)	−3.628	<0.001 *
RBC (*10^12^ /L)	4.4 (3.91,4.84)	4.66 (4.33,5.09)	4.37 (3.88,4.81)	−3.975	<0.001*
NE (%)	63 (55.9,69.8)	62.34 (55.1,69.1)	63.05 (56,69.875)	−0.432	0.7
EO (%)	1.5 (0.655,2.8)	1.4 (0.4,2.8)	1.5 (0.7,2.8)	−0.804	0.4
LY (%)	27.8 (20.65,33.8)	28.02 (21.575,34.7)	27.64 (20.5,33.7)	−0.982	0.3
MO (%)	5.7 (4.5,7.3)	5.75 (4.7,7.875)	5.7 (4.5,7.2)	−0.959	0.3
IgG (g/L)	9.09 (6.2175,11.8)	5.415 (3.1725,6.3575)	9.375 (6.79,12.0225)	−8.922	<0.001 *
IgA (g/L)	2.51 (1.87,3.32)	2.185 (1.735,2.945)	2.54 (1.88,3.34)	−2.276	0.02 *
IgM (g/L)	1.06 (0.72,1.48)	1.44 (0.9632,1.88)	1.03 (0.7068,1.45)	−4.187	<0.001 *
C3 (g/L)	1.07 (0.907,1.24)	1.19 (1.0575,1.425)	1.06 (0.897,1.23)	−4.443	<0.001 *
C4 (g/L)	0.243 (0.1942,0.31)	0.272 (0.2253,0.3403)	0.24 (0.19,0.309)	−3.136	0.002 *
IgE (IU/ml)	52.2 (19.4142)	160 (46.7982)	47.3 (19,126)	−5.890	<0.001 *
C1q (g/L)	0.308 (0.266,0369)	0.369 (0.305,0.4205)	0.304 (0.263,0.3622)	−5.092	<0.001 *
Urine protein (mg/24 h)	2425 (1008.95004.2)	5284.62 (3786.54,9330.5)	2179.46 (973.4425,4599)	−7.4	< 0.001*
eGFR (ml/min)	101.065 (66.68,124.035)	115.59 (82.23,135)	100.17 (65.07,123.48)	−2.458	0.014*
Immunosuppressive therapy					
YES		**66**	**548**		
NO		**5**	**388**		
Missing data			**2**		

*P < 0.05 MCD group vs. non-MCD group

*Abbreviations*: *SBP* Systolic blood pressure, *DBP* Diastolic blood pressure, *BMI* Body mass index, *Glu* Glucose, *WBC* White blood count, *HB* Hemoglobin, *PLT* Platelet, *RBC* Red blood cell count, *NE* Neutrophil percentage, *EO* Eosinophil percentage, *LY* Lymphocyte percentage, *MO* Monocyte percentage, *IgG* Immunoglobulin G, *IgA* Immunoglobulin A, *IgM* Immunoglobulin M, *C3* Complement 3, *C4* Complement 4, *IgE* Immunoglobulin E, *C1q* Complement 1q

### Logistic LASSO regression analysis and ROC analysis

**Fig. 1:**
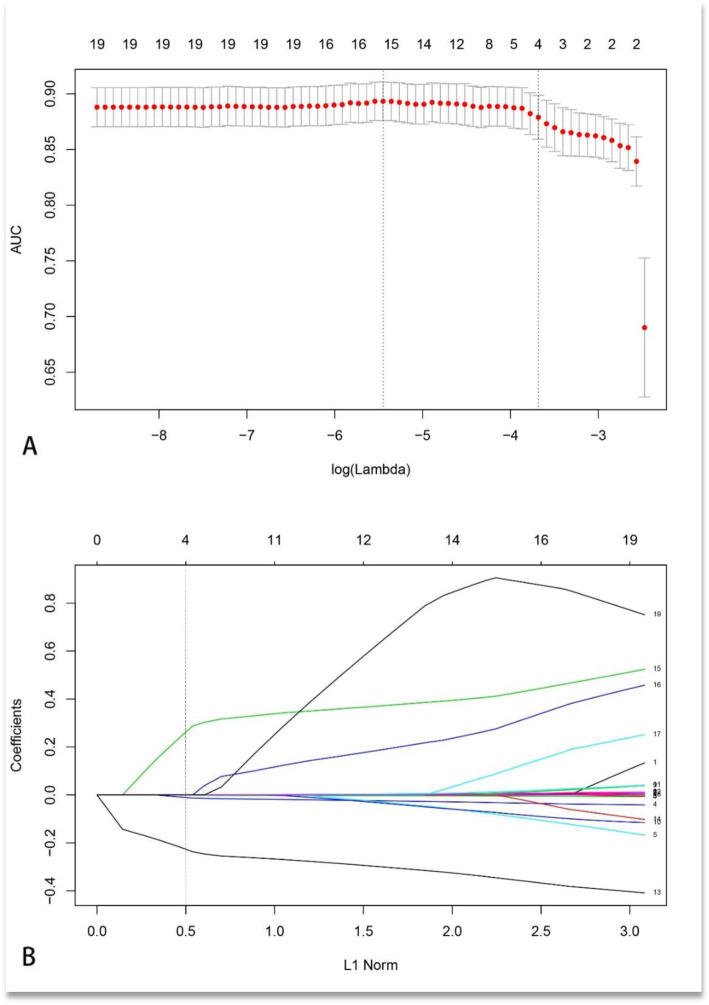
Construction of the MCD diagnosis model. **a** Statistics of the ROC curves for the 23 associated parameters between MCD and non-MCD. Two vertical lines are drawn at the values (i.e., 4 and 16) chosen by cross-validation. **b** LASSO coefficient profiles of the 23 MCD-associated characteristics. A vertical line is drawn at the value chosen by cross-validation

LASSO is a popular method used in regression analysis with high-dimensional predictors [15]. Before initially establishing the model, as many independent variables as possible are usually selected to minimize the model deviation due to a lack of important independent variables. In this way, the 25 variables in our study were all substituted into the modelling procedure. The predicted probabilities (PRE-1) were calculated and saved. Among these 25 variable candidates, DBP and blood IgG, IgM, and IgE were suggested to be significant predictors of an MCD diagnosis (Fig. 1b). We noticed that the AUC gradually increased as the number of variables increased, which were used to establish the diagnostic prediction model. Cross-validation was then performed on the lambda grid points, and the lambda value with the smallest cross-validation error was selected (i.e., either 4 or 16 variables).

**Fig. 2:**
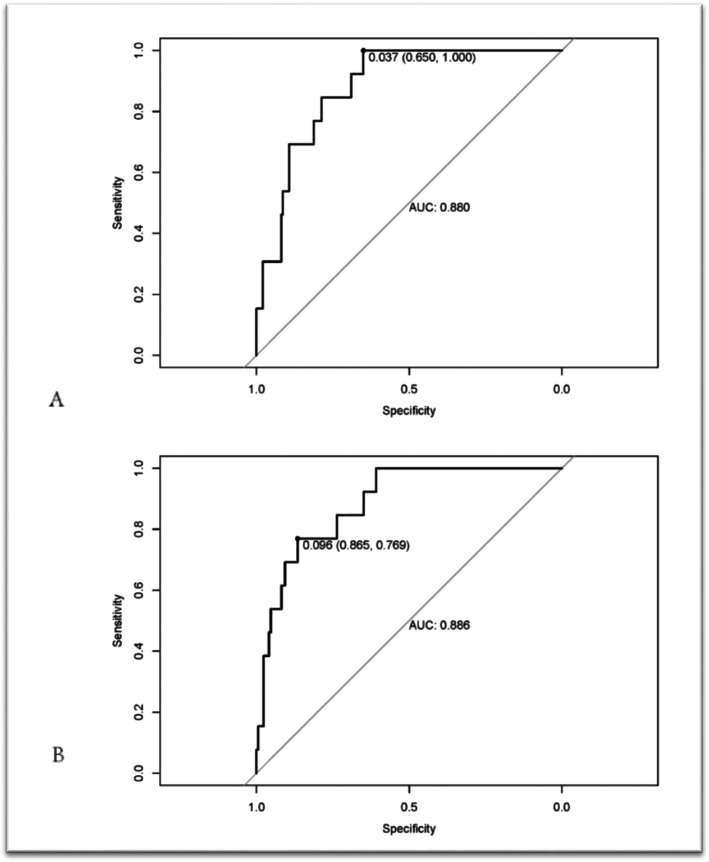
ROC curves of the two MCD classification models. **a** ROC curve for the “DBP+ IgG+ IgM+ IgE” combination. **b** ROC curve for the 16-parameter model

However, the modelling process needs to find the independent variable set that has the strongest explanatory power to the dependent variable, that is, to improve the interpretability and prediction accuracy of the model through independent variable selection. We calculated the AUC of the two models and plotted the ROC graphs (Fig. 2a and b). According to the principle of preventing overfitting, the “DBP + IgG + IgM + IgE” combination was strikingly significant in distinguishing between MCD and non-MCD, which was evaluated via logistic LASSO regression analysis. The AUCs for determining MCD in the combined model consisting of “DBP + IgG + IgM + IgE” and in the 16 parameter-based model were 0.88 and 0.886, respectively (Fig. 2). Finally, according to the obtained lambda value, we refit the model with all the data.

### Nomogram for predicting the risk of MCD

To provide nephrologists with a quantitative method to predict a patient’s probability of having MCD while avoiding an unnecessary renal biopsy, we constructed a nomogram based on the validation data set and the equation from the discriminant analysis.

**Fig. 3:**
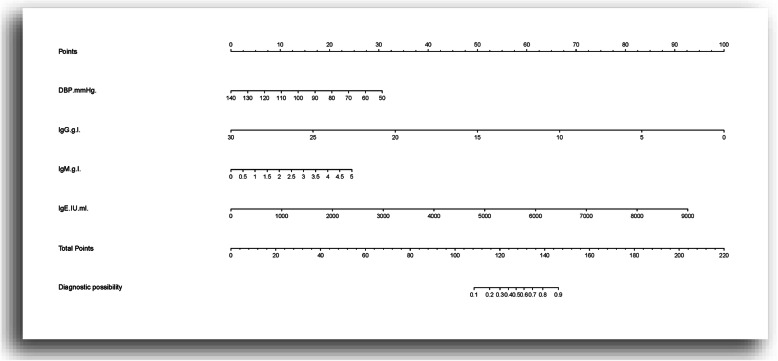
Nomogram for predicting the risk of MCD

It can be seen from Fig. 3 that the composition of the nomogram can be divided into three categories: a) Variables used in the prediction model, for example, DBP and IgG. The line segment corresponding to each variable is marked with a scale, which represents the range of possible values of the variable, and the length of the line segment reflects the outcome of the factor, that is, the size of the contribution of an event; b) The corresponding variable scores, namely, the points at the top of the figure, which represent the corresponding scores of each variable for different values. The total score of the corresponding individual scores after all the variables are added is the Total Points; c) Prediction of the probability of the occurrence of an event: For example, the line “risk” at the bottom of the figure represents the probability of the occurrence of MCD.

## Discussion

In the present study, we established and validated a novel prognostic tool based on the “DBP + IgG + IgM + IgE” combination to improve the prediction of an MCD diagnosis and avoiding an unnecessary biopsy. This proposed model can successfully predict the probability of an MCD diagnosis in patients with NS.

The present study was designed as a retrospective cohort study. Compared with previous studies, our study included more parameters, including C3, C4 and C1q, etc., all of which are known as important biomarkers for common glomerular diseases with NS. As such, the model tool established from these parameters could be more powerful and accurate for the prediction of MCD diagnosis [16, 17]. Based on statistical analysis, the current understanding of the mechanisms of MCD development and practical clinical experience, 12 out of 25 useful parameters were selected as predictors for MCD diagnosis. The 12 parameters included SBP, DBP, HB, PLT, RBC, IgG, IgA, IgM, C3, C4, IgE, and C1q. Among them, four indicators, including DBP, IgG, IgM and IgE, were specifically screened. Similar results were obtained by using commonly used multiparameter analyses and logistic LASSO regression analyses, which confirmed that these four parameters were strikingly significant in differentiating an MCD from a non-MCD diagnosis. Furthermore, 25% of patient data were used to validate the derived equations for classifying MCD.

Recently published studies demonstrated that blood pressure variability (BPV) is considered an important cardiovascular disease (CVD) risk factor, with evidence suggesting that it is associated with clinical outcomes [18–21]. The results reported by Sethna et al. showed that the application of the association of BPV with renal outcomes could be extended to patients with primary glomerulopathy. Moreover, they further described a 5% increase in the occurrence of the composite endpoint (ESRD or eGFR decline < 40%) with a one-unit increase in the SD of SBP [22]. Aggressively lowering systolic blood pressure which lowers diastolic pressure to less than 70 mmHg may increase mortality risk for patients with chronic kidney disease according to an observational study [23]. Given these clinical observations, we speculate that this slight difference in blood pressure between MCD patients and non-MCD patients might affect the clinical outcome and is possibly clinically significant. We will continue to study the effect of blood pressure in this population of patients.

The exact pathogenesis of MCD has not yet been well elucidated. Previous studies have shown that the serum IgE might play an important role in the pathogenesis of MCD and might serve as a prognostic indicator in terms of steroid responsiveness in MCD patients [24, 25]. There is a large body of evidence to suggest that T cells, especially type 2 helper T cells, play a key role in driving podocyte injury in MCD [26]. Of all cytokines released by Th2 cells, it is frequently associated with increased levels of interleukin-4 (IL-4) and raised production of IL-13. IL-13 regulates the switching of immunoglobulin production toward IgE [27–29]. Several studies also revealed increased serum IL-13 levels in patients with MCD [30]. This finding is consistent with our finding for the performance of increased serum IgE levels in predicting MCD. The predictive value of serum IgE for MCD diagnosis has gradually become accepted in recent years [31].

Other studies have revealed that the levels of serum IgG and IgM decrease during the relapse of steroid-sensitive nephrotic syndrome [32–34]. Disproportionally decreased levels of IgG subclasses, especially of IgG1 and IgG2, cause a decrease in serum total IgG levels during relapses [33, 35]. Decreased serum IgG levels may result from urinary loss of IgG or an impaired class switch from IgM to IgG in B cells [33]. Our study showed a significantly decreased IgG level in patients with MCD and an increased serum IgM level, as expected. Although some studies have shown the glomerular deposition of these immunoglobulins in kidney specimens of MCD patients [25, 34, 36, 37], they seemed more likely to be non-specific makers of any glomerular disease due to potential blood immunoglobulin contamination in the glomerular capillaries, especially of IgG or IgM. Based on the papers published available, there are few studies on the relationship between these parameters and pathological indicators reflecting the severity of the disease.

We realize that there are some limitations in our study. This was a retrospective cohort study, and it is critical to further verify the sensitivity and specificity of the model for the diagnosis of MCD in clinical practice. The model was established based on data from patients of Chinese ethnicity, especially adults, so its generalizability is limited, and it may be susceptible to the causes of the inherent biases of such a study format. We will further validate the accuracy and repeatability of the prediction model for an MCD diagnosis with prospective studies in multicentre clinical trials.

Few clinical risk prediction models for an MCD diagnosis have been developed. Combining multiple factors (DBP, IgG, IgM and IgE) displayed a better effect in predicting the diagnosis of MCD than that of prior reports [16, 17, 38]. The measurement of serum immunoglobulins is cheaper and faster than that of other indexes and is easily available in general hospitals. Furthermore, we utilized a more scientific statistical method to avoid the interference of human factors on the construction of the model and randomly selected a subset of patient data to internally test our model.

## Conclusions

In summary, we first established a diagnostic model that combined multiple factors (DBP, IgG, IgM and IgE) to effectively distinguish MCD patients from non-MCD patients among adults with high sensitivity and specificity. The four parameter-based classifier potentially offers clinical value in predicting a patient’s probability of having MCD diagnosis, avoiding renal biopsy in adults with NS.

## Data Availability

The datasets analyzed during the current study are available from the corresponding author on reasonable request.

## Abbreviations

MCD: Minimal change disease
NS: Nephrotic syndrome
DBP: Diastolic blood pressure
AUC: Area under the curve
MsPGN: Mesangial proliferative glomerulonephritis
FSGS: Focal segmental glomerulosclerosis
MN: Membranous nephropathy
MPGN: Membranoproliferative glomerulonephritis
ROC: Receiver operating characteristic
SD: Standard deviation
SBP: Systolic blood pressure
HB: Hemoglobin
PLT: Platelet
RBC: Red blood cell count
IgG: Immunoglobulin G
IgA: Immunoglobulin A
IgM: Immunoglobulin M
C3: Complement 3
C4: Complement 4
IgE: Immunoglobulin E
C1q: Complement 1q
BPV: Blood pressure variability
CVD: Cardiovascular disease
BMI: Body mass index
Glu: Glucose
WBC: White blood count
NE: Neutrophil percentage
EO: Eosinophil percentage
LY: Lymphocyte percentage
MO: Monocyte percentage

## References

[1] Shimada M, Araya C, Rivard C, Ishimoto T, Johnson RJ, Garin EH (2011). Minimal change disease: a ‘two-hit’ podocyte immune disorder?. Pediatr Nephrol.

[2] Vivarelli M, Massella L, Ruggiero B, Emma F (2017). Minimal change disease. Clin J Am Soc Nephrol.

[3] Eddy AA, Symons JM. Nephrotic syndrome in childhood. Lancet. 2003. 10.1016/S0140-6736(03)14184-0. 10.1016/S0140-6736(03)14184-0 12944064

[4] Floege J, Amann K (2016). Primary glomerulonephritides. Lancet.

[5] Nachman PH, Jennette JC, Falk RJ (2011). “Primary glomerular disease,” in Brenner and Rector’s the kidney.

[6] Cameron JS (1987). The Nephrotic syndrome and its complications. Am J Kidney Dis.

[7] Waldman M (2007). Adult Minimal-Change Disease: Clinical Characteristics, Treatment, and Outcomes. Clin J Am Soc Nephrol.

[8] Fisi V (2012). Histological diagnosis determines complications of percutaneous renal biopsy: a single-center experience in 353 patients. Kidney Blood Press Res.

[9] Mohamed N, John R (2011). Use of renal biopsy in the elderly. Int Urol Nephrol.

[10] Whittier WL, Korbet SM (2004). Renal biopsy: Update. Curr Opinion Nephrol Hypertens.

[11] Steyerberg EW, Eijkemans MJC, Harrell FE, Habbema JDF (2001). Prognostic modeling with logistic regression analysis: in search of a sensible strategy in small data sets. Med Decis Mak.

[12] Stiglic G (2015). Comprehensible predictive modeling using regularized logistic regression and comorbidity based features. PLoS One.

[13] Tibshirani R. Regression shrinkage and selection via the lasso. J R Stat Soc Ser B. 1996. 10.1111/j.2517-6161.1996.tb02080.x.

[14] Hajian-Tilaki K. “Receiver operating characteristic (ROC) curve analysis for medical diagnostic test evaluation,”. Caspian J Intern Med. 2013. PMC3755824 24009950

[15] Tibshirani R. Regression shrinkage and selection via the lasso: a retrospective. J R Stat Soc Ser B Stat Methodol. 2011. 10.1111/j.1467-9868.2011.00771.x.

[16] Hsiao CC (2018). Immunoglobulin e and G Levels in Predicting Minimal Change Disease before Renal Biopsy. Biomed Res Int.

[17] Shao YN (2009). Serum immunoglobulin e can predict minimal change disease before renal biopsy. Am J Med Sci.

[18] Rothwell PM (2010). Prognostic significance of visit-to-visit variability, maximum systolic blood pressure, and episodic hypertension. Lancet.

[19] Mallamaci F (2013). Long-term visit-to-visit office blood pressure variability increases the risk of adverse cardiovascular outcomes in patients with chronic kidney disease. Kidney Int.

[20] Whittle J (2016). Visit-to-visit variability of BP and CKD outcomes: results from the ALLHAT. Clin J Am Soc Nephrol.

[21] Diaz KM (2014). Visit-to-visit variability of blood pressure and cardiovascular disease and all-cause mortality a systematic review and meta-analysis. Hypertension.

[22] Sethna CB (2017). Blood pressure and visit-to-visit blood pressure variability among individuals with primary Proteinuric Glomerulopathies. Hypertension.

[23] Kovesdy CP (2013). Blood pressure and mortality in U.S. veterans with chronic kidney disease: a cohort study. Ann Intern Med.

[24] Shu KH, Lian JD, Yang YF, Lu YS, Wang JY (1988). Serum IgE in primary glomerular diseases and its clinical significance. Nephron.

[25] Groshong T, Mendelson L, Mendoza S, Bazaral M, Hamburger R, Tune B (1973). Serum IgE in patients with minimal-change nephrotic syndrome. J Pediatr.

[26] Bacharier LB, Geha RS (2000). Molecular mechanisms of IgE regulation. J Allergy Clin Immunol.

[27] Lai KW (2007). Overexpression of interleukin-13 induces minimal-change-like nephropathy in rats. J Am Soc Nephrol.

[28] Parry RG, Gillespie KM, Mathieson PW (2001). Effects of type 2 cytokines on glomerular epithelial cells. Exp Nephrol.

[29] Cheung W, Wei CL, Seah CC, Jordan SC, Yap HK (2004). Atopy, serum IgE, and interleukin-14 in steroid-responsive nephrotic syndrome. Pediatr Nephrol.

[30] Kimata H, Fujimoto M, Furusho K (1995). Involvement of interleukin (IL)-13, but not IL-4, in spontaneous IgE and IgG4 production in nephrotic syndrome. Eur J Immunol.

[31] Tan Y, Yang D, Fan J, Chen Y (2011). Elevated levels of immunoglobulin E may indicate steroid resistance or relapse in adult primary nephrotic syndrome, especially in minimal change nephrotic syndrome. J Int Med Res.

[32] Chan MK, Chan KW, Jones B (1987). Immunoglobulins (IgG, IgA, IgM, IgE) and complement components (C3, C4) in nephrotic syndrome due to minimal change and other forms of glomerulonephritis, a clue for steroid therapy?. Nephron.

[33] Kemper MJ, Altrogge H, Ganschow R, Muüller-Wiefel DE (2002). Serum levels of immunoglobulins and IgG subclasses in steroid sensitive nephrotic syndrome. Pediatr Nephrol.

[34] Yamada N (2018). Relationship between immunoglobulin deposition and early lesions of progressive Glomerulonephropathy in young common marmosets. Vet Pathol.

[35] Mishra OP, Garg R, Usha ZA, Das BK (1997). Immunoglobulins and circulating immune complexes in nephrotic syndrome. J Trop Pediatr.

[36] Dong J, Peng T, Gao J, Jia X, Yan G, Wang Y (2018). A pilot and comparative study between pathological and serological levels of immunoglobulin and complement among three kinds of primary glomerulonephritis. BMC Immunol.

[37] Takei T (2007). The characteristics of relapse in adult-onset minimal-change nephrotic syndrome. Clin Exp Nephrol.

[38] Ling C, et al. Urinary CD80 levels as a diagnostic biomarker of minimal change disease. Pediatr Nephrol. 2014. 10.1007/s00467-014-2915-3. 10.1007/s00467-014-2915-3 25142334

